# Cardiac Remodeling from Middle Age to Senescence

**DOI:** 10.3389/fphys.2017.00341

**Published:** 2017-05-26

**Authors:** Mikko J. Möttönen, Olavi Ukkola, Jarmo Lumme, Y. Antero Kesäniemi, Heikki V. Huikuri, Juha S. Perkiömäki

**Affiliations:** Research Unit of Internal Medicine, Medical Research Center Oulu, Oulu University Hospital, University of OuluOulu, Finland

**Keywords:** aging, cardiac remodeling, left ventricular size, left ventricular mass, left ventricular walls, fractional shortening, left atrial diameter

## Abstract

**Background:** The data on cardiac remodeling outside the scope of myocardial infarction and heart failure are limited.

**Methods:** A cohort of middle-aged hypertensive subjects with age- and gender-matched control subjects without hypertension (*n* = 1,045, aged 51 ± 6 years) were randomly selected for the OPERA study (Oulu Project Elucidating Risk of Atherosclerosis study). The majority of those who were still alive after more than 20 years of follow-up underwent thorough re-examinations.

**Results:** Left ventricular mass index (LVMI) increased significantly from 106.5 ± 27.1 (mean ± SD) to 114.6 ± 29.1 g/m^2^ (*p* < 0.001), the thickness of the left ventricular posterior wall (LVPW) from 10.0 ± 1.8 to 10.6 ± 1.7 mm (*p* < 0.001), fractional shortening (FS) from 35.0 ± 5.7 to 38.4 ± 7.2 % (*p* < 0.001), and left atrial diameter (LAD) from 38.8 ± 5.2 to 39.4 ± 6.7 mm (*p* = 0.028) during the 20-year follow-up. After multivariate adjustments, hypertension treated with antihypertensive medication and male gender predicted a smaller increase in the thickness of LVPW (*p* = 0.017 to <0.001). Baseline higher fasting plasma insulin level, larger intima media thickness of the carotid artery, greater height and antihypertensive medication (*p* = 0.046–0.002) predicted a smaller (less favorable) change of FS. The increase of LAD was associated with higher baseline diastolic blood pressure (*p* = 0.034) and greater height (*p* = 0.006).

**Conclusion:** Aging from middle age to senescence increases the echocardiographic indexes of LVMI, LVPW thickness, FS and LAD. Several baseline factors are associated with these changes.

## Introduction

Cardiac remodeling refers to a series of changes at the molecular and cellular level resulting in a progressive physiological and anatomical transformation, such as left ventricular dilatation, hypertrophy and ultimately, changes in left ventricle geometry from a more elliptical shape toward a spherical globe (Cohn et al., 2000; Katz and Zile, 2006; Gajarsa and Kloner, 2011). The left atrium is also susceptible to structural remodeling, characterized by increase in size and volume (Abhayaratna et al., 2006; Leung et al., 2008). Cardiac remodeling may not show clinical symptoms until after several years of progression. Adverse cardiac remodeling is typically caused by an injury to the myocardium, such as acute myocardial infarction, but it also occurs in response to pressure overload (hypertension, aortic stenosis), in inflammatory heart muscle disease, nonischemic dilated cardiomyopathy and volume overload (valvular regurgitation), and frequently progresses to heart failure (Remes, 1994; Cohn et al., 2000; Muhlfeld et al., 2013). Ventricular remodeling may lead to changes such as cardiomyocyte lengthening, myocyte hypertrophy or loss, accumulation of collagen in the cardiac interstitium, ventricular wall thinning, infarct expansion and scar formation (Cohn et al., 2000). Left atrial pressure and volume overload, present in heart failure and other pathological states, have been associated with left atrial dilatation (Abhayaratna et al., 2006; Leung et al., 2008). Neurohormonal changes, mainly the activation of the renin–angiotensin–aldosterone system, which has been intensively studied, as well as beta-adrenergic regulation are seen to drive the cellular-level changes, which eventually lead to remodeling (Remes, 1994; Cohn et al., 2000; Fukuta and Little, 2007). Most of the current understanding of cardiac remodeling comes from studies on patients suffering from myocardial infarction (Cohn et al., 2000; Muhlfeld et al., 2013). The current data on cardiac remodeling outside the scope of myocardial infarction and heart failure are thus limited. Therefore, we aimed to assess cardiac remodeling/normal adaptation and to identify the baseline factors that are associated with the cardiac remodeling in a prospective study in which subjects aged from 40 to 59 years without history of myocardial infarction or heart failure were followed up for more than 20 years.

## Methods

### Study population and design

The Oulu Project Elucidating Risk of Atherosclerosis (OPERA) study is a prospective, population-based, epidemiological study designed to address the risk factors and disease end points of atherosclerotic cardiovascular diseases. A total of 1,045 middle-aged subjects (aged 51 ± 6 years (mean ± SD), age range from 40 to 59 years) were initially recruited to the study between the years 1990 and 1993. The study population consisted of 520 men (261 hypertensives and 259 controls) and 525 women (258 hypertensives and 267 controls). Hypertensive subjects were randomly selected from the national register for reimbursement of medication. Age- and gender-matched control subjects were selected randomly from the national health register excluding any subjects with the right to reimbursement for hypertension medication. The subjects with a history of myocardial infarction or significant heart disease were not included in the study. Details of the study population have been described previously (Rantala et al., 1999). The study subjects, *n* = 1,004 after exclusions, went through thorough clinical examinations at baseline including standardized blood pressure measurements, laboratory tests, an assessment of autonomic cardiac regulation and echocardiographic studies. Validated questionnaires were used to determine alcohol consumption and smoking history. The majority of the 813 subjects who were still alive after more than 20 years of follow-up were willing to participate in re-examinations and the subjects with baseline and follow-up echocardiographic studies (*n* = 552) were included in the present analysis. This study was carried out in accordance with the recommendations of the guidelines of the Ethical Committee of the Faculty of Medicine, University of Oulu with written informed consent from all subjects. All subjects gave written informed consent in accordance with the Declaration of Helsinki. The protocol was approved by the Ethical Committee of the Faculty of Medicine, University of Oulu.

### Blood pressure measurements

The blood pressure measurements were performed according to the recommendations of the American Society of Hypertension. An automatic oscillometric blood pressure recorder (Dinamap, Critikon Ltd) was used to measure the blood pressure according to a standardized protocol three times at 1-min intervals. The mean value of the second and third blood pressure measurement was used in the analyses (Rantala et al., 1999).

### Echocardiographic examinations

At baseline, echocardiographic measurements were performed by the same experienced cardiologist, blinded to patients' clinical data, using a Hewlett-Packard 77020A ultrasound color system. Details of the methodology have been described elsewhere (Airaksinen et al., 1986, 1989). The measurements were based on the recommendations of the American Society of Echocardiography (Airaksinen et al., 1989). Left ventricular mass was calculated using the formula of Troy (Troy et al., 1972). Left ventricular mass index (LVMI) was determined by dividing the left ventricular mass by the body surface area. Fractional shortening (FS) was calculated as [left ventricular internal diastolic diameter (LVIDD)—left ventricular internal systolic diameter (LVISD)]/LVIDD and used as a measure of left ventricular systolic function and remodeling (Cohn et al., 2000). The FS values below the range 26–28% for European males, and 26–27% for European females are considered abnormal (Echocardiographic Normal Ranges Meta-Analysis of the Left Heart Collaboration., 2015). At the follow-up, the echocardiographic examinations were performed in a core lab using a GE Healthcare Vivid E 9 VERSION 110. x.x ultrasound system. The measurements were determined according to the recommendations of the American Society of Echocardiography (Lang et al., 2015).

### Laboratory tests

All the laboratory test samples were obtained after an overnight fast. Blood was drawn to EDTA tubes and plasma was separated by centrifugation and stored for analysis. Several routine laboratory analyses were conducted as described earlier (Rantala et al., 1999).

### Carotid artery ultrasonographies

A single trained radiologist performed the carotid artery ultrasonographies according to the same protocol using a duplex ultrasound system with a 7.5 MHz scanning frequency in B-mode, pulsed-Doppler mode and color mode (Päivänsalo et al., 1996). Intima-media thickness (IMT) was measured at five locations on each side. The mean IMT was determined according to a standardized protocol.

### Statistical analysis

The paired-samples *T*-test was used to assess the statistical significance of the change from baseline to follow-up visit in LVIDD (ΔLVIDD), interventricular septum (ΔIVS), left ventricular posterior wall (ΔLVPW), in LVMI (ΔLVMI), FS (ΔFS), and left atrial diameter (ΔLAD). The statistical significance of differences in echocardiographic parameters and in their change between the subjects with hypertension and the controls was assessed using the standard *t*-test. ΔLVIDD, ΔIVS, ΔLVPW, ΔLVMI, ΔFS, and ΔLAD were divided into tertiles. The statistical significance of differences of baseline and follow-up parameters between the tertiles was evaluated using the one-way analysis of variance for continuous variables and the χ^2^-test for categorical variables. The independent power of the parameters which differed between the tertiles at the *p* < 0.1 level and had a significant bivariate linear correlation with the delta-values was assessed in the multivariate linear regression analysis model using backward elimination. Data were analyzed using the IBM SPSS Statistics software 22.0 (Armonk, NY: IBM Corp.). A *p*-value < 0.05 was considered to be statistically significant.

## Results

### Cardiac remodeling during the follow-up

LVIDD tended to increase in the subjects with hypertension, but did not change significantly in the controls during more than 20 years of follow-up (Table 1). The thickness of IVS and LVPW increased significantly in the controls, as did LVPW in the subjects with hypertension during the follow-up. There was a significant regression of the thickness of IVS in the subjects with hypertension during the follow-up. Left ventricular walls were significantly thicker in the subjects with hypertension when compared with the controls both at baseline and at the follow-up visit. During the follow-up, LVMI increased significantly both in the controls and the subjects with hypertension. The LVMI in the subjects with hypertension was higher than in the controls both at baseline and at the follow-up visit. FS increased significantly in both the controls and the subjects with hypertension during the follow-up. The subjects with hypertension had significantly higher FS at baseline, but not at the follow-up visit when compared with the controls. The increase in LAD was significant in the overall study population, but did not reach statistical significance in the controls or the subjects with hypertension. The subjects with hypertension had a significantly larger left atrium compared with the controls both at baseline and at the follow-up visit (Table 1). FS increased significantly more in controls during the follow-up when compared with the subjects with hypertension (Table 2). There was a significantly larger change in the thickness of ventricular walls in the controls during the follow-up when compared with the subjects with hypertension (Table 2).

**Table 1:** **Cardiac remodeling during more than 20 years of follow-up**.

**Parameter**	**At baseline**	**At follow-up**	***p*-value**
**LVIDD, mm**			
-All study subjects	51.6 ± 4.9	51.9 ± 6.4	0.13
-Controls	51.3 ± 4.8	51.5 ± 6.6	0.68
-Hypertension	51.9 ± 5.0	52.5 ± 6.1	0.08
**IVS, mm**			
-All study subjects	10.6 ± 2.2	10.7 ± 2.3	0.60
-Controls	10.1 ± 1.9	10.4 ± 2.7	0.039
-Hypertension	11.3 ± 2.2^***^	11.0 ± 1.8^**^	0.049
**LVPW, mm**			
-All study subjects	10.0 ± 1.8	10.6 ± 1.7	< 0.001
-Controls	9.5 ± 1.8	10.3 ± 1.6	< 0.001
-Hypertension	10.6 ± 1.8^***^	10.9 ± 1.8^***^	0.004
**LVMI, g/m**^2^			
-All study subjects	106.5 ± 27.1	114.6 ± 29.1	< 0.001
-Controls	100.8 ± 24.7	110.1 ± 28.8	< 0.001
-Hypertension	113.3 ± 28.3^***^	120.0 ± 28.5^***^	0.001
**FS, %**			
-All study subjects	35.0 ± 5.7	38.4 ± 7.2	< 0.001
-Controls	34.4 ± 5.7	38.8 ± 7.1	< 0.001
-Hypertension	35.8 ± 5.5^**^	37.9 ± 7.3	< 0.001
**LAD, mm**			
-All study subjects	38.8 ± 5.2	39.4 ± 6.7	0.028
-Controls	37.6 ± 5.0	38.2 ± 6.9	0.11
-Hypertension	40.3 ± 5.2^***^	40.9 ± 6.2^***^	0.14

*The values are presented as mean ± SD. All study subjects: n = 552; controls: n = 295; hypertension (subjects with hypertension): n = 257. FS, fractional shortening; IVS, interventricular septum; LAD, left atrial diameter; LVIDD, left ventricular internal diastolic diameter; LVMI, left ventricular mass index; LVPW, left ventricular posterior wall*.

*** *p < 0.001*,

** *p < 0.01 between controls and the subjects with hypertension*.

**Table 2:** **Change of echocardiographic parameters during more than 20 years of follow-up**.

**Parameter**	**Controls (*n* = 295)**	**Subjects with hypertension (*n* = 257)**	***p*-value**
Delta LVIDD, mm	0.13 ± 5.5	0.62 ± 5.7	0.30
Delta IVS, mm	0.36 ± 3.0	−0.28 ± 2.3	0.005
Delta LVPW, mm	0.83 ± 1.9	0.37 ± 2.1	0.006
Delta LVMI, g/m^2^	9.3 ± 29.3	6.7 ± 30.4	0.33
Delta FS, %	4.5 ± 7.8	2.1 ± 7.8	< 0.001
Delta LAD, mm	0.6 ± 6.2	0.6 ± 5.9	0.96

*The values are presented as mean ± SD. Delta = the change of the value of a parameter between baseline and follow-up visit. The abbreviations are the same as in the Table 1*.

### Association of baseline factors with cardiac remodeling

**Figure 1:**
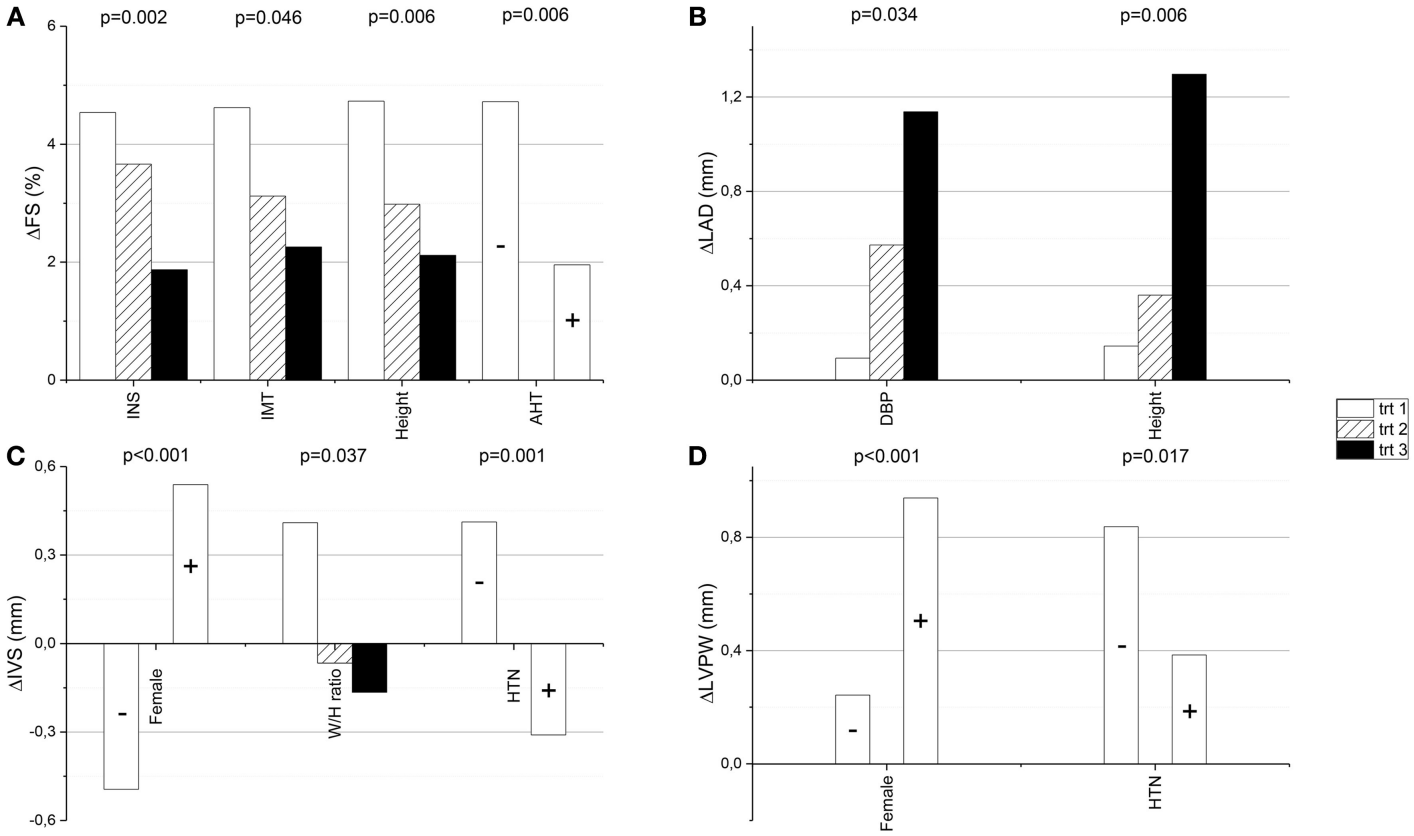
**The change of fractional shortening (ΔFS), left atrial diameter (ΔLAD), interventricular septum (ΔIVS) and left ventricular posterior wall (ΔLVPW) from the baseline to the follow-up visit are presented in relation to tertiles (or presence/absence) of the baseline factors, which had a significant association with the change in multivariate linear regression analysis model (the *p*-values are from this model** ; **A–D**, respectively). AHT, antihypertensive medication (+ = present, − = absent); DBP, diastolic blood pressure; HTN, hypertension (+ = present, − = absent); IMT, the intima-media thickness of carotid artery; INS, fasting plasma insulin; trt, tertile and W/H ratio, waist-to-hip ratio.

The baseline factors that differed significantly between the tertiles of ΔFS and ΔLAD are shown in Tables 3, 4, respectively. None of the baseline factors shown in Tables 3, 4 differed significantly between the tertiles of ΔLVMI or had a significant linear association with the increase in LVIDD (data not shown). Baseline higher fasting plasma insulin level, larger IMT of the carotid artery, greater height and antihypertensive medication had a significant association with smaller ΔFS in the multivariate linear regression analysis model (Table 5, Figure 1A). Based on the β values, 46.3% of the change in FS could be explained by these baseline factors. Higher baseline diastolic blood pressure and larger height were still significantly associated with the increase in LAD after multivariate adjustments in the linear regression analysis (Table 5, Figure 1B). These factors explained 21.8% of the change in LAD. Larger ΔIVS and ΔLVPW were significantly associated with female sex, while smaller ΔIVS and ΔLVPW were significantly associated with hypertension with treatment at baseline, and ΔIVS also with waist/hip ratio in the multivariate model (Table 5, Figures 1C,D). The aforementioned baseline factors explained 57.1% of the change in IVS and 26% of the change in LVPW.

**Table 3:** **Association of baseline factors with change in fractional shortening after more than 20 years of follow-up**.

**Baseline variables**	**1st trt of ΔFS**	**2nd trt of ΔFS**	**3rd trt of ΔFS**	***p*-value**
Age, years	50.3 ± 6.1	50.4 ± 5.4	49.5 ± 5.4	0.28
Gender, female	46%	55%	60%	0.030
Height, cm	169.0 ± 8.8	168.7 ± 8.5	166.6 ± 9.1	0.023
Weight, kg	81.3 ± 15.0	75.8 ± 13.4	73.9 ± 14.8	< 0.001
Body mass index, kg/m^2^	28.4 ± 4.3	26.6 ± 3.9	26.5 ± 4.1	< 0.001
Waist, cm	92.2 ± 12.2	87.7 ± 11.6	85.9 ± 12.1	< 0.001
Waist/Hip ratio	0.87 ± 0.08	0.85 ± 0.08	0.84 ± 0.08	0.001
Smoking (pack-years)	8.8 ± 12.8	6.4 ± 11.8	6.6 ± 10.6	0.10
Alcohol consumption (g/week)	64.2 ± 81.1	44.2 ± 66.3	47.1 ± 56.6	0.12
Systolic blood pressure, mmHg	147.3 ± 19.3	145.3 ± 21.4	141.2 ± 19.2	0.013
Diastolic blood pressure, mmHg	88.9 ± 11.3	87.8 ± 12.1	85.8 ± 11.5	0.043
Pulse pressure, mmHg	58.5 ± 13.4	57.6 ± 13.8	55.4 ± 12.9	0.081
eGFR, mL/min/1.73 m^2^	80.4 ± 15.3	81.8 ± 16.3	83.4 ± 15.8	0.21
Atrial natriuretic peptide, pg/ml	279 ± 161	252 ± 132	256 ± 111	0.13
Fasting plasma insulin, mmol/l	13.9 ± 9.6	10.6 ± 5.7	10.7 ± 6.3	< 0.001
Fasting plasma glucose, mmol/l	4.8 ± 1.4	4.5 ± 1.2	4.4 ± 0.9	0.013
HDL-cholesterol, mmol/l	1.30 ± 0.36	1.40 ± 0.39	1.43 ± 0.41	0.006
LDL-cholesterol, mmol/l	3.53 ± 0.95	3.50 ± 0.87	3.35 ± 0.86	0.12
Triglycerides, mmol/l	1.52 ± 0.79	1.43 ± 0.76	1.36 ± 0.82	0.14
hs-CRP, mg/l	4.0 ± 9.7	2.9 ± 4.8	2.2 ± 4.1	0.045
IMT, mm	0.83 ± 0.12	0.82 ± 0.13	0.79 ± 0.11	0.015
Diabetes	13 (7%)	12 (7%)	7 (4%)	0.35
CAD	16 (9%)	6 (3%)	8 (4%)	0.042
Hypertension	106 (59%)	86 (47%)	75 (41%)	0.003
Antihypertensive medication	110 (61%)	84 (46%)	73 (40%)	< 0.001
-Beta blockers	64 (36%)	43 (23%)	37 (20%)	0.002
-ACE-inhibitors	33 (18%)	32 (17%)	32 (18%)	0.97
-Diuretics	40 (22%)	19 (10%)	19 (10%)	0.001
-Calcium channel blockers	24 (13%)	16 (9%)	14 (8%)	0.16
-Other antihypertensive medication	5 (3%)	10 (5%)	4 (2%)	0.20

*The change in fractional shortening (ΔFS) was divided into tertiles (1st trt < 1.0%, 2nd trt ≥ 1.0% but < 6.8%, 3rd trt ≥ 6.8%). The 1st tertile denotes the least favorable change. ACE, angiotensin converting enzyme; CAD, coronary artery disease; eGFR, estimated glomerular filtration rate; FS, fractional shortening; HDL-cholesterol, high-density lipoprotein cholesterol; hs-CRP, high sensitivity C-reactive protein; IMT, intima media thickness of carotid artery; LDL-cholesterol, low-density lipoprotein cholesterol; trt, tertile*.

**Table 4:** **Association of baseline factors with change in left atrial diameter after more than 20 years of follow-up**.

**Baseline variables**	**1st trt of ΔLAD**	**2nd trt of ΔLAD**	**3rd trt of ΔLAD**	***p*-value**
Age, years	50.7 ± 5.6	49.1 ± 5.5	50.3 ± 5.8	0.025
Gender, female	56%	59%	46%	0.041
Height, cm	167.2 ± 8.5	167.2 ± 8.9	170.1 ± 9.0	0.002
Weight, kg	76.9 ± 14.1	75.7 ± 15.6	77.5 ± 14.2	0.51
Body mass index, kg/m^2^	27.4 ± 4.1	27.0 ± 4.7	26.6 ± 3.5	0.25
Waist, cm	88.6 ± 12.5	87.7 ± 12.8	88.4 ± 11.6	0.80
Waist/Hip ratio	0.85 ± 0.08	0.84 ± 0.09	0.86 ± 0.09	0.44
Smoking (pack-years)	7.3 ± 12.4	7.4 ± 11.8	7.2 ± 11.4	0.98
Alcohol consumption (g/week)	48.5 ± 66.6	49.2 ± 71.7	57.5 ± 67.4	0.42
Systolic blood pressure, mmHg	142.4 ± 18.4	144.4 ± 20.5	146.4 ± 21.7	0.19
Diastolic blood pressure, mmHg	85.6 ± 12.0	87.3 ± 11.8	89.4 ± 11.2	0.014
Pulse pressure, mmHg	56.8 ± 11.9	57.1 ± 13.8	57.1 ± 14.6	0.97
eGFR, mL/min/1.73 m^2^	80.9 ± 17.2	81.5 ± 13.9	82.6 ± 16.7	0.63
Atrial natriuretic peptide, pg/ml	255 ± 118	267 ± 133	274 ± 162	0.45
Fasting plasma insulin, mmol/l	11.4 ± 6.9	11.7 ± 8.5	11.1 ± 6.3	0.77
Fasting plasma glucose, mmol/l	4.4 ± 0.5	4.4 ± 0.5	4.4 ± 0.5	0.54
HDL-cholesterol, mmol/l	1.36 ± 0.38	1.40 ± 0.40	1.41 ± 0.41	0.34
LDL-cholesterol, mmol/l	3.43 ± 0.84	3.45 ± 0.86	3.51 ± 0.98	0.69
Triglycerides, mmol/l	1.45 ± 0.77	1.34 ± 0.66	1.44 ± 0.88	0.31
hs-CRP, mg/l	3.4 ± 9.1	2.3 ± 4.6	3.2 ± 6.3	0.29
IMT, mm	0.82 ± 0.12	0.80 ± 0.11	0.8 ± 0.12	0.13
Diabetes	1 (1%)	2 (1%)	2 (1%)	0.86
CAD	9 (5%)	7 (4%)	13 (8%)	0.17
Hypertension	84 (47%)	92 (50%)	71 (46%)	0.79
Antihypertensive medication	82 (46%)	89 (48%)	76 (49%)	0.84
-Beta blockers	53 (30%)	48 (26%)	36 (23%)	0.41
-ACE-inhibitors	27 (15%)	33 (18%)	30 (19%)	0.57
-Diuretics	23 (13%)	25 (13%)	22 (14%)	0.92
-Calcium channel blockers	13 (7%)	16 (9%)	18 (12%)	0.35
-Other antihypertensive medication	4 (2%)	6 (3%)	7 (5%)	0.50

*The change in left atrial diameter (ΔLAD) was divided into tertiles (1st trt < −2.0 mm, 2nd trt ≥ −2.0 mm but < 3.0 mm, 3rd trt ≥ 3.0 mm). The 3rd tertile stands for the least favorable change. The abbreviations are described in Table 3*.

**Table 5:** **Association of baseline factors with change in echocardiographic parameters in multivariable model**.

**Echocardiographic parameter**	**Baseline variables**	**β**	***p*-value**	***R*^2^**
Δ**FS**				
	Fasting plasma insulin level	−0.136	0.002	0.086
	IMT of the carotid artery	−0.086	0.046	0.086
	Height	−0.120	0.006	0.086
	Antihypertensive medication	−0.121	0.006	0.086
Δ**LAD**				
	Diastolic blood pressure	0.095	0.034	0.030
	Height	0.123	0.006	0.030
Δ**IVS**				
	Female sex	0.284	<0.001	0.058
	Hypertension with treatment	−0.148	0.001	0.058
	Waist/hip ratio	0.139	0.037	0.058
Δ**LVPW**				
	Female sex	0.159	0.001	0.038
	Hypertension with treatment	−0.101	0.017	0.038

*The baseline factors, which retained a significant association with the change of the echocardiographic parameter after adjustments in the multivariable linear regression analysis model, are shown in the table. ΔFS, the change of fractional shortening, ΔIVS, the change of the thickness of interventricular septum, ΔLAD, the change of left atrial diameter, ΔLVPW, the change of the thickness of left ventricular posterior wall during the follow-up, IMT, intima-media thickness*.

### Association of follow-up factors with cardiac remodeling

**Figure 2:**
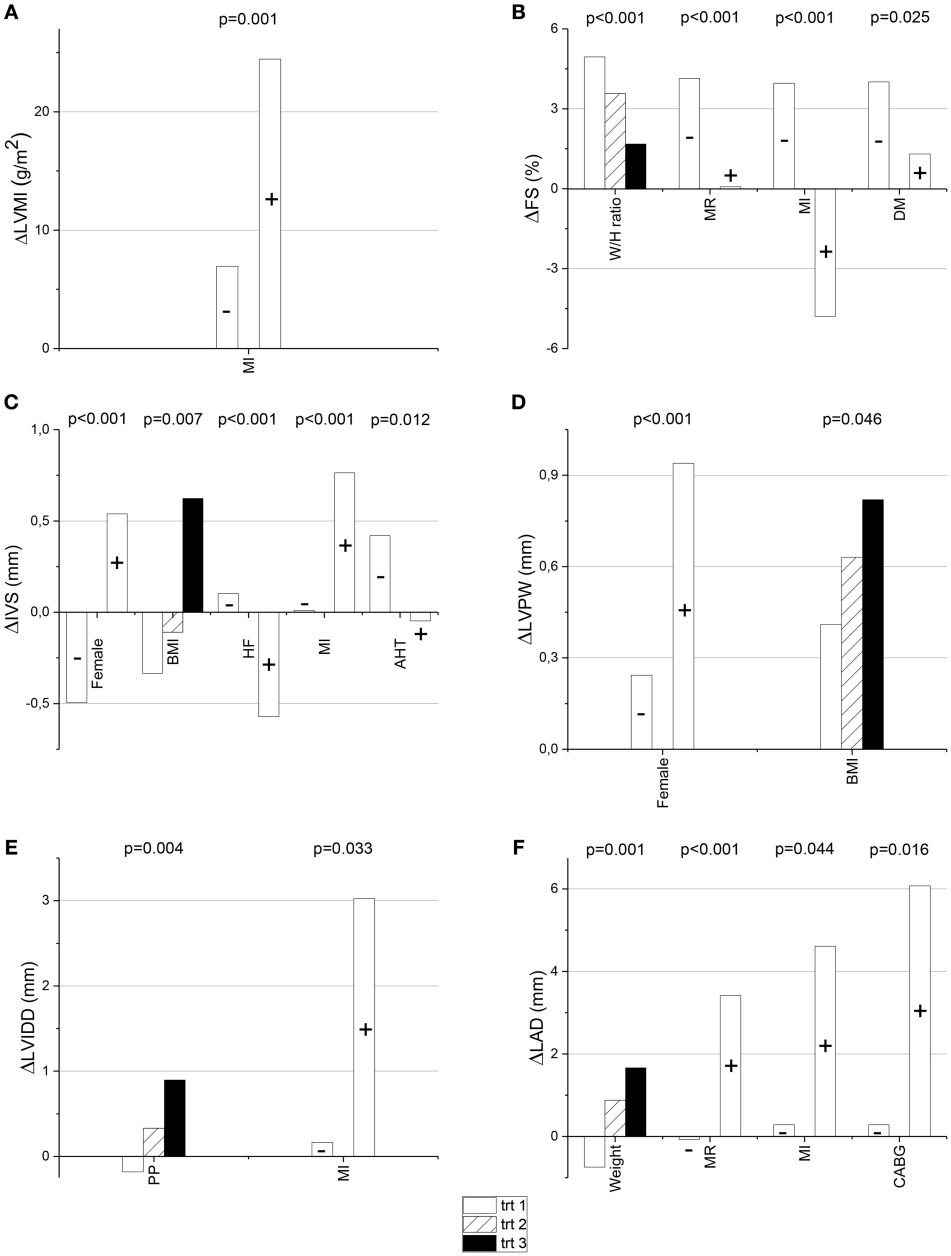
**The change of left ventricular mass index (ΔLVMI), fractional shortening (ΔFS), interventricular septum (ΔIVS), left ventricular posterior wall (ΔLVPW), left ventricular internal diastolic diameter (ΔLVIDD), and left atrial diameter (ΔLAD) from the baseline to the follow-up visit are presented in relation to tertiles (or presence/absence) of the follow-up factors, which had a significant association with the change in multivariate linear regression analysis model (the *p*-values are from this model; A–F**, respectively). AHT, antihypertensive medication (+ = present, − = absent); BMI, body mass index; CABG, coronary artery bypass grafting (+ = present, − = absent); DM, diabetes mellitus (+ = present, − = absent); HF, heart failure (+ = present, − = absent); MI, myocardial infarction (+ = present, − = absent); MR, mitral regurgitation (+ = present, − = absent); PP, pulse pressure, trt, tertile and W/H ratio, waist-to-hip ratio.

By the time of the follow-up visit, a proportion of the study subjects had developed cardiac and other diseases. Evidence of myocardial infarction was found in 6.9% of the subjects, 4.9% had undergone a coronary artery bypass grafting procedure, and 6.2% a percutaneous coronary intervention. Of the study subjects, 20.3% had coronary artery disease, 23.4% diabetes, 19.0% mitral regurgitation, 15.4% aortic regurgitation, 1.3% aortic stenosis, and 1.3% evidence of heart failure. Evidence of previous myocardial infarction was the only follow-up factor which had a significant univariate and multivariate association with the increase of LVMI in the multivariate linear regression analysis model (Table 6, Figure 2A). Larger waist-to-hip ratio, presence of mitral regurgitation, previous myocardial infarction and diabetes had a significant association with smaller ΔFS even after adjustments for other risk indicators (Table 6, Figure 2B). Female gender was also significantly associated with larger ΔIVS and ΔLVPW when adjusted for relevant follow-up factors. Larger ΔIVS and ΔLVPW also had a significant multivariate association with body mass index, larger ΔIVS with the evidence of previous myocardial infarction, and smaller ΔIVS with the use of antihypertensive medication and a history of heart failure (Table 6, Figures 2C,D). Of the follow-up parameters, higher pulse pressure and previous myocardial infarction had a significant association with the increase of LVIDD after adjusting for all the relevant risk indicators (Table 6, Figure 2E). When the follow-up factors were tested in the multivariable linear regression analysis model, higher weight, mitral regurgitation, previous myocardial infarction, and a history of coronary artery bypass grafting had a significant association with the increase in LAD (Table 6, Figure 2F).

**Table 6:** **Association of Follow-up factors with change in echocardiographic parameters in multivariable model**.

**Echocardiographic parameter**	**Follow-up variables**	**β**	***p*-value**	***R*^2^**
Δ**LVMI**				
	Previous myocardial infarction	0.148	0.001	0.034
Δ**FS**				
	Waist-to-hip ratio	−0.154	0.001	0.144
	Mitral regurgitation	−0.197	0.001	0.144
	Previous myocardial infarction	−0.229	<0.001	0.144
	Diabetes	−0.092	0.025	0.144
Δ**IVS**				
	Female sex	0.213	<0.001	0.111
	Body mass index	0.115	0.007	0.111
	Previous myocardial infarction	0.163	<0.001	0.111
	Antihypertensive medication	−0.107	0.012	0.111
	History of heart failure	−0.194	<0.001	0.111
Δ**LVPW**				
	Female sex	0.171	<0.001	0.037
	Body mass index	0.017	0.046	0.037
Δ**LVIDD**				
	Pulse pressure	0.122	0.004	0.043
	Previous myocardial infarction	0.104	0.033	0.043
Δ**LAD**				
	Weight	0.145	0.001	0.116
	Mitral regurgitation	0.217	<0.001	0.116
	Previous myocardial infarction	0.097	0.044	0.116
	History of CABG	0.121	0.012	0.116

*The follow-up factors, which retained a significant association with the change of the echocardiographic parameter after adjustments in the multivariable linear regression analysis model, are shown in the table. ΔFS, the change of fractional shortening, ΔIVS, the change of the thickness of interventricular septum, ΔLAD, the change of left atrial diameter, ΔLVIDD, the change of left ventricular internal diastolic diameter, ΔLVMI, the change of left ventricular mass index, ΔLVPW, the change of the thickness of left ventricular posterior wall during the follow-up, CABG, coronary artery bypass grafting*.

## Discussion

During the aging of the present study subjects, cardiac remodeling/adaptation manifested as an increase in left ventricular mass and increase in the degree of shortening of left ventricular diameter between end-diastole and end-systole both in the subjects with hypertension and the controls. The thickness of ventricular walls increased in controls and the thickness of LVPW also in the subjects with hypertension. The subjects with hypertension had larger left ventricular mass, left atrial size, and thicker ventricular walls in comparison with the controls both at baseline and at the follow-up visit, showing that hypertension modifies cardiac morphology and remodeling. The increase in FS and in the thickness of ventricular walls was larger in the controls compared with the subjects with hypertension. Although we were unable to evaluate the change of the shape of the left ventricle, it is plausible to speculate that the subjects with hypertension had more frequently geometrical changes in the left ventricle than the controls during the follow-up. This may have led to the remodeling and relative thinning of ventricular walls and decrease in FS more commonly in the subjects with hypertension compared with the controls (Cohn et al., 2000). This concept is supported by our observation that the left ventricular size tended to increase in the subjects with hypertension but not in the controls during the follow-up. The thickness of IVS even regressed in the subjects with hypertension, which may also partly be explained by antihypertensive medication. Several components of metabolic syndrome at baseline were associated with changes in FS, higher fasting plasma concentrations of insulin being an independent predictor of less favorable change in FS. Female gender was associated with the thickening of ventricular walls and hypertension with treatment with the regression of the thickness of ventricular walls. The increase in left atrial size was predicted by larger height and higher diastolic blood pressure. Several disease states present at the time of the follow-up visit, such as a previous myocardial infarction, were associated with cardiac remodeling.

Most of the data on cardiac remodeling come from studies in patients with history of myocardial infarction. After myocardial infarction, remodeling occurs in both the infarcted and non-infarcted myocardium, and the degree of remodeling is directly proportional to the infarct size (Pfeffer and Braunwald, 1990; Chareonthaitawee et al., 1995). Adverse cardiac remodeling occurs also in response to other pathologic states, such as hypertension, heart failure, increased afterload caused by stiffening of the arteries or aortic stenosis, and volume overload caused by both mitral and aortic regurgitation (Remes, 1994; Cohn et al., 2000; Muhlfeld et al., 2013). Compatible with these notions, at the time of the follow-up visit evidence of previous myocardial infarction was associated with the increase of left ventricular mass and size, thickening of IVS, less favorable change in FS and increase in left atrial size. Mitral regurgitation causing volume overload was associated with less favorable change in FS and increase in left atrial size, whereas a history of heart failure was associated with a decrease in the thickness of IVS, which may be explained by geometrical changes leading to a more spherical left ventricle in heart failure (Cohn et al., 2000; Gajarsa and Kloner, 2011). Furthermore, larger pulse pressure at the time of the follow-up visit was associated with the increase in left ventricular size during the follow-up.

FS provides a measurement of the contractile function of the left ventricle, and it is also used as a measurement of cardiac remodeling (de Simone et al., 1994; Cohn et al., 2000). In the present study, overall FS remained well above the abnormal range, and even increased during the follow-up. It has been reported that FS calculated from ventricular diameters may overestimate myocardial function when ventricular wall thickness increases (Shimizu et al., 1991; de Simone et al., 1994). This may be the explanation for the increase of FS in the present population during aging. It is logical that the components of metabolic syndrome as risk factors of atherosclerosis contributed to a less favorable change in FS. The importance of atherosclerosis in this process is emphasized by our observation that larger IMT at baseline predicted a less favorable change in FS. During the follow-up, a proportion of the study subjects had developed cardiac and other diseases. At the time of the follow-up visit, evidence of prior myocardial infarction, mitral regurgitation, diabetes mellitus, and larger waist/hip ratio were associated with less favorable change in FS, further supporting the important influence of atherosclerosis and its risk factors on left ventricular function/remodeling during aging. Larger body mass index measured at the follow-up visit was associated with the thickening of ventricular walls, an observation which is also compatible with the concept that the metabolic syndrome is involved in the cardiac remodeling process during aging.

Increase in left atrial size has commonly been noted being a response to atrial pressure and volume overload. Pressure overload occurs in mitral stenosis or in increasing ventricular filling pressures, and volume overload in mitral regurgitation (Abhayaratna et al., 2006; Leung et al., 2008). Therefore, it is logical that higher baseline diastolic blood pressure predicted the increase in left atrial size. At baseline, the study subjects were aged from 40 to 59 years. At this age, the diastolic component of hypertension is relatively more prominent whereas during aging, arterial stiffening leads to increase in systolic and decrease in diastolic blood pressure (O'Rourke, 1971; Emoto et al., 1998). The association of larger baseline height with the increase of left atrial size can partly be explained by the observation that the male study subjects tended to have a larger increase in left atrial size than the females. The aging alone does not independently contribute to left atrial enlargement and it is not seen as part of the normal aging process (Pearlman et al., 1990; Thomas et al., 2003). Age-related left atrial enlargement is rather associated with pathological processes contributing to the enlargement. This concept is compatible with our present findings showing that at the time of the follow-up visit, evidence of previous myocardial infarction, a history of coronary artery bypass grafting, mitral regurgitation, and larger weight were associated with the increase of left atrial size.

Our present study has some limitations. There was a notable drop-out of the study subjects as a proportion of the subjects had died or were not willing to participate in the follow-up examinations. We were unable to assess the influence of the observed morphological remodeling on adverse cardiovascular events and mortality as this data will be gathered during further follow-up after the follow-up visit. In the present analysis, we were also unable to evaluate detailed changes in the remodeling, such as the change in the shape of the left ventricle. It is noteworthy that in the present population resembling a general population, many individual changes of left ventricular mass, size and wall thicknesses, left atrial size and FS occurred in the normal range and presumably represent normal adaptation rather than adverse remodeling.

In conclusion, our analysis yields unique information on the morphological cardiac remodeling and adaptation during aging in a population which was initially aged from 40 to 59 years.

## Author contributions

Each author has contributed significantly to the submitted work. MM: participated in the conception and design of the study and in the analysis and interpretation of data, wrote the manuscript, and approved the submission of the manuscript. OU: contributed to the conception and design of the study and collection of the data, revised the manuscript critically, and approved the manuscript submission. JL: participated in the examination of the study subjects, contributed to the conception of the study, revised the manuscript, and approved the manuscript submitted. YAK: participated in the collection and analyses of the study population, interpretation of data, revised the manuscript, and approved the submitted manuscript. HH: participated in the conception and design of the study and in the analysis and interpretation of data, revised the manuscript critically for important intellectual content, and approved the submission of the manuscript. JP: participated in the conception and design of the study and in the analysis and interpretation of data, drafting and revising of the manuscript, and approved the submission of the manuscript.

### Conflict of interest statement

The authors declare that the research was conducted in the absence of any commercial or financial relationships that could be construed as a potential conflict of interest.

## References

[B1] Abhayaratna W. P. Seward J. B. Appleton C. P. Douglas P. S. Oh J. K. Tajik A. J.. (2006). Left atrial size: physiologic determinants and clinical applications. J. Am. Coll. Cardiol. 47, 2357–2363. 10.1016/j.jacc.2006.02.048 16781359

[B2] Airaksinen K. E. Ikaheimo M. J. Salmela P. I. Kirkinen P. Linnaluoto M. K. Takkunen J. T. (1986). Impaired cardiac adjustment to pregnancy in type I diabetes. Diabetes Care 9, 376–383. 10.2337/diacare.9.4.376 3527613

[B3] Airaksinen K. E. Koistinen M. J. Ikaheimo M. J. Huikuri H. V. Korhonen U. Pirttiaho H.. (1989). Augmentation of atrial contribution to left ventricular filling in IDDM subjects as assessed by Doppler echocardiography. Diabetes Care 12, 159–161. 10.2337/diacare.12.2.159 2702899

[B4] Chareonthaitawee P. Christian T. F. Hirose K. Gibbons R. J. Rumberger J. A. (1995). Relation of initial infarct size to extent of left ventricular remodeling in the year after acute myocardial infarction. J. Am. Coll. Cardiol. 25, 567–573. 10.1016/0735-1097(94)00431-O 7860898

[B5] Cohn J. N. Ferrari R. Sharpe N. (2000). Cardiac remodeling–concepts and clinical implications: a consensus paper from an international forum on cardiac remodeling. Behalf of an International forum on cardiac remodeling. J. Am. Coll. Cardiol. 35, 569–582. 10.1016/s0735-1097(99)00630-0 10716457

[B6] de Simone G. Devereux R. B. Roman M. (1994). Assessment of left ventricular function by the midwall fractional shortening/end-systolic stress relation in human hypertension. J. Am. Coll. Cardiol. 23, 1441–1451. 10.1016/0735-1097(94)90390-5 8176105

[B7] Echocardiographic Normal Ranges Meta-Analysis of the Left Heart Collaboration. (2015). Ethnic-specific normative reference values for echocardiographic, L. A., and LV Size, LV Mass, and systolic function: the echonormal study. JACC Cardiovasc. Imaging 8, 656–665. 10.1016/j.jcmg.2015.02.014 25981507

[B8] Emoto M. Nishizawa Y. Kawagishi T. Maekawa K. Hiura Y. Kanda H.. (1998). Stiffness indexes beta of the common carotid and femoral arteries are associated with insulin resistance in NIDDM. Diabetes Care 21, 1178–1182. 10.2337/diacare.21.7.1178 9653616

[B9] Fukuta H. Little W. C. (2007). Contribution of systolic and diastolic abnormalities to heart failure with a normal and a reduced ejection fraction. Prog. Cardiovasc. Dis. 49, 229–240. 10.1016/j.pcad.2006.08.009 17185111

[B10] Gajarsa J. J. Kloner R. A. (2011). Left ventricular remodeling in the post-infarction heart: a review of cellular, molecular mechanisms, and therapeutic modalities. Heart Fail. Rev. 16, 13–21. 10.1007/s10741-010-9181-7 20623185

[B11] Katz A. M. Zile M. R. (2006). New molecular mechanism in diastolic heart failure. Circulation 113, 1922–1925. 10.1161/CIRCULATIONAHA.106.620765 16636184

[B12] Lang R. M. Badano L. P. Mor-Avi V. Afilalo J. Armstrong A. Ernande L.. (2015). Recommendations for cardiac chamber quantification by echocardiography in adults: an update from the american society of echocardiography and the european association of cardiovascular imaging. Eur. Heart J. Cardiovasc. Imaging 16, 233–270. 10.1093/ehjci/jev014 25712077

[B13] Leung D. Y. Boyd A. Ng A. A. Chi C. Thomas L. (2008). Echocardiographic evaluation of left atrial size and function: current understanding, pathophysiologic correlates, and prognostic implications. Am. Heart J. 156, 1056–1064. 10.1016/j.ahj.2008.07.021 19032999

[B14] Muhlfeld C. Schipke J. Schmidt A. Post H. Pieske B. Sedej S. (2013). Hypoinnervation is an early event in experimental myocardial remodelling induced by pressure overload. J. Anat. 222, 634–644. 10.1111/joa.12044 23565587 PMC3666243

[B15] O'Rourke M. F. (1971). The arterial pulse in health and disease. Am. Heart J. 82, 687–702. 10.1016/0002-8703(71)90340-1 4940223

[B16] Päivänsalo M. Rantala A. Kauma H. Lilja M. Reunanen A. Savolainen M.. (1996). Prevalence of carotid atherosclerosis in middle-aged hypertensive and control subjects. A cross-sectional systematic study with duplex ultrasound. J. Hypertens. 14, 1433–1439. 10.1097/00004872-199612000-00008 8986926

[B17] Pearlman J. D. Triulzi M. O. King M. E. Abascal V. M. Newell J. Weyman A. E. (1990). Left atrial dimensions in growth and development: normal limits for two-dimensional echocardiography. J. Am. Coll. Cardiol. 16, 1168–1174. 10.1016/0735-1097(90)90549-5 2229763

[B18] Pfeffer M. A. Braunwald E. (1990). Ventricular remodeling after myocardial infarction. Experimental observations and clinical implications. Circulation 81, 1161–1172. 10.1161/01.CIR.81.4.1161 2138525

[B19] Rantala A. O. Kauma H. Lilja M. Savolainen M. J. Reunanen A. Kesaniemi Y. A. (1999). Prevalence of the metabolic syndrome in drug-treated hypertensive patients and control subjects. J. Intern. Med. 245, 163–174. 10.1046/j.1365-2796.1999.00429.x 10081519

[B20] Remes J. (1994). Neuroendocrine activation after myocardial infarction. Br. Heart J. 72, S65–S69. 10.1136/hrt.72.3_Suppl.S65 7946807 PMC1025596

[B21] Shimizu G. Hirota Y. Kita Y. Kawamura K. Saito T. Gaasch W. H. (1991). Left ventricular midwall mechanics in systemic arterial hypertension. Myocardial function is depressed in pressure-overload hypertrophy. Circulation 83, 1676–1684. 10.1161/01.CIR.83.5.1676 1827056

[B22] Thomas L. Levett K. Boyd A. Leung D. Y. C. Schiller N. B. Ross D. L. (2003). Changes in regional left atrial function with aging: evaluation by Doppler tissue imaging. Eur. J. Echocardiogr. 4, 92–100. 10.1053/euje.4.2.92 12749870

[B23] Troy B. L. Pombo J. Rackley C. E. (1972). Measurement of left ventricular wall thickness and mass by echocardiography. Circulation 45, 602–611. 10.1161/01.CIR.45.3.602 4258936

